# A New Approach for Combining Time-of-Flight and RGB Cameras Based on Depth-Dependent Planar Projective Transformations

**DOI:** 10.3390/s150924615

**Published:** 2015-09-23

**Authors:** Carlota Salinas, Roemi Fernández, Héctor Montes, Manuel Armada

**Affiliations:** 1 Centre for Automation and Robotics (CAR) CSIC-UPM, Ctra. Campo Real, Km. 0,200, La Poveda. Arganda del Rey, Madrid 28500, Spain; E-Mails: roemi.fernandez@car.upm-csic.es (R.F.); hector.montes@ csic.es (H.M.); manuel.armada@csic.es (M.A.); 2 Facultad de Ingeniería Eléctrica, Universidad Tecnológica de Panamá, Panamá City 0819, Panama

**Keywords:** high resolution RGB-D maps, depth-dependent homography lookup table, image registration, uncalibrated methods

## Abstract

Image registration for sensor fusion is a valuable technique to acquire 3D and colour information for a scene. Nevertheless, this process normally relies on feature-matching techniques, which is a drawback for combining sensors that are not able to deliver common features. The combination of ToF and RGB cameras is an instance that problem. Typically, the fusion of these sensors is based on the extrinsic parameter computation of the coordinate transformation between the two cameras. This leads to a loss of colour information because of the low resolution of the ToF camera, and sophisticated algorithms are required to minimize this issue. This work proposes a method for sensor registration with non-common features and that avoids the loss of colour information. The depth information is used as a virtual feature for estimating a depth-dependent homography lookup table (*Hlut*). The homographies are computed within sets of ground control points of 104 images. Since the distance from the control points to the ToF camera are known, the working distance of each element on the *Hlut* is estimated. Finally, two series of experimental tests have been carried out in order to validate the capabilities of the proposed method.

## 1. Introduction

3D imaging is a crucial stage for a worthwhile variety of robotic fields such as robot navigation, precision agriculture, robot assistance, and many others [1]. There are many active and passive techniques for capturing the information of the 3D world. Some of them are based on time-of-flight (ToF) cameras, laser scanning, stereovision system and pattern projection (structured light). All of these techniques have different uses in robotics applications, as well as their advantages and weaknesses, but all of them provide more or less accurate information for reconstructing surfaces [2,3]. 

This work is focused on techniques for sensor registration that provide 3D information (structure and colour) for dynamically changing environments, which means either the robot is in motion or objects in the scene are in motion. Therefore, fast algorithms that qualify for near real-time conditions are desired. In such a scenario, laser-based methods are non-suitable solutions for real-time applications, because normally, they require moving parts to scan the scene row by row. The same is true of structured light methods, where besides the scanning of light projection, 3D sensing also needs to be carried out under very controlled light conditions. Passive camera-based methods including depth from motion, shape and focus and stereo triangulation normally require solving the correspondence problem. The first group of methods involves acquiring multiple images, which produces ambiguities and singularities, and this introduces additional computation load and temporal cost. On the other hand, the stereo triangulation technique is the most common and well-known technique for acquiring 3D information. Its working principle is to determine what pair of points on two images are the corresponding projections of a same 3D point. A very interesting review of these systems is presented by Scharstein and Szeliski [4]. Over the last two decades, significant improvements have been made for solving the correspondence problem. Nevertheless, the problem of occlusion mismatching and the incapability of matching textureless regions remains unsolved. 

Alternatively, ToF cameras are becoming more and more popular, less expensive and more powerful. Some interesting works have evaluated these cameras, and they have shown their advantages in certain fields. In conclusion, the most relevant attribute of these systems is their capability of delivering simultaneously depth maps and intensity images at a video frame rate. However, their spatial resolution is very low, not more than thousands of pixels are provided, and they tend to be noisy and poorly calibrated. In [5] the authors presented a methodology to reduce the errors in the depth measurements of the SR4000 camera [6]. They modelled the measurement errors with a sinusoidal approximation and calibrated the intrinsic camera parameters. A most extensive evaluation of the ToF cameras was presented by Foix *et al.* [7]. This work shows the potential of these systems, but due to their limited resolution they conclude that previous technologies are still leading the 3D sensing field. However, the combination of ToF and colour cameras has shown great improvements to compensate for this lack of resolution. A comparative study between the stereo vision systems and ToF cameras is beyond the scope of this work, and interested readers may refer to Beder *et al.* [8].

The approaches for combining ToF and colour cameras are commonly presented in two configurations: the monocular setting which combines a single colour camera and a ToF camera, and the coupling of a stereo vision system and a ToF camera. In this work a monocular setup is adopted. In general, the fusion of these two systems is addressed by computing the extrinsic parameters for the homogenous transformation between them, which means that the method efficiency relies on the cameras calibration and the accuracy of the depth measurements of the ToF camera. In the stereo configuration, the 3D-3D correspondences are used to estimate the transformation between the two systems. Some approaches utilize the depth from the ToF as a constraint to compute the stereo matching. In [9,10], the authors use fast algorithms and inaccurate extrinsic parameters to improve the disparity computation, and results show the sensor fusion is possible. However, when upscaling the dense maps to the colour image size, some problems at the objects’ edges are reported, and only visual results are reported. On the other hand, very interesting results are presented in [11], where the authors calibrate the system within a range of 400 mm and use the depth values as an additional observed variable in the global approximation function. In this case, the method was tested in real scenes and numerical results report mean errors within 2–3 pixels. Nevertheless, this approach assumes a global regularization method for stereo matching, which normally is not fast enough for real time applications [4]. Regardless, in this configuration the most important drawbacks of the stereo system, which are the occlusions and textureless regions, remain unsolved.

In the monocular case, the depth information is used during the calibration process to back-project the 3D points into the 2D points of the RGB image. Normally, in related works, orthogonal generation is applied for the cameras frames co-alignment [12]. However, other researches adopted projective texturing. In this case, the RGB camera is projected onto the ToF camera projective geometry. Unfortunately, in both cases, only few works present numeric results of their methods implementation [13,14]. As it was mentioned in Foix, Alenya and Torras [7], the challenging issue is how to handle the difference between the cameras’ resolutions, because between each pair of nearby points of the ToF image there are several points of the colour image. Therefore, the complexity of this work lies in the upsampling techniques for computing high-resolution depth maps without losing the colour information and achieving near real-time processing conditions. Most of the related works upscale depth maps of up to 1.5 Megapixels by means of bilinear or bicubic interpolation [15]. However, the proposal of this work deals with high resolution colour dense maps of 5 Megapixels. Other approaches are concerned with improving the quality of the high resolution depth maps. For instance, in [16], the authors present an interpolation algorithm for edge enhancement that uses the gradient and Laplacian to adjust the sampling location, but only visual results of a single object scene are presented. Remarkable efforts have been made in [14] to create high-accuracy depth maps, where outlier detection was addressed as a minimization function of the Mark Random field. Then through a robust optimization function that combines several factors, namely the data, the neighborhood smoothness and the non-local mean regularization, depth fusion was achieved. Their results stands out from other algorithms, but the complexity of the method makes it unreliable for real-time applications. The authors report a computation time for real-world scenes of 19.00 s. A similar case is presented in Huhle *et al*. [17], where a GPU implementation for parallel computation is adopted, with the aim of implementing a denoising and enhancement filter based on non-local means formulation. In this case it takes nearly 2 s to complete the processing. 

As is mentioned above, the ToF and RGB sensor fusion relies on the extrinsic calibration and the depth estimations from the ToF camera. The depth information is noisy and because of the ToF camera’s low resolution, the extrinsic parameters are inaccurate. In some cases it is possible to achieve good results without accurate extrinsic calibration as is shown in [10]. Other works report some simplification when sensors are mounted in particular configurations [15,18,19,20]. The typical noise of the depth measurements can be modelled as a Poisson distribution around the true value. However, the artifacts derived from the object’s albedo are not easy to model. Most of the related works addressed the problem by filtering techniques of the depth values. Nevertheless, the filtering can often over-smooth the interpolated data, significantly affecting the depth discontinuities of the boundaries. In the search of a method for registering sensors that deliver data with non-common features, and are additionally capable of addressing biased depth measurements, the proposal in this work undertakes the idea of working with uncalibrated methods for automatic data registration, which has not been studied yet. 

The distinction of the uncalibrated methods is that they do not first need to know the internal and external parameters of the cameras. This may normally lead to a system capable of achieving up to projective reconstruction. Nevertheless, the theory introduced by Hartley and Zisserman [21], regarding multiple view geometry, demonstrates the possibility of achieving both affine and Euclidean reconstruction with no previous knowledge of the camera calibration matrix.

Regarding projective geometry, there are two relations between two views (cameras) and a scene plane. The first relation is the epipolar geometry, which represents the intersection of a pencil with two image planes, where the axis of the pencil is a line joining the cameras’ centres, denoted as the baseline. The intersection of the baseline with the image planes are the epipoles (e, e′). Given that information, it is possible to back-project an image point x on image 1 to a ray in the 3-space. The ray passes through the camera centre, the point x and the 3-space point *X*, which is on one plane of the pencil. This ray is projected onto image 2 as a line that intersects its epipole (e′). Then, the problem of finding a correspondence for x, which is the projection of *X* on image 2, is reduced to a search on a line. The second relation is given through the plane projective transformation, which is the relation of image points on a plane in a view to the corresponding image points in a second view by a planar homography, $\;x_{2}=Hx_{1}$.

Consequently, when considering the search for corresponding points in a 3D-space scene, epipolar geometry is the straightforward solution to reckon with. Nonetheless, the problems of feature matching based on images are very well known. Most of the problems arise from the occlusions and the changes in the illumination conditions, and all of them contribute to non-matched or wrongly matched features. Some works have presented solutions for these problems, such as the method introduced by Sagüés [22], where the authors propose matching lines between images instead of matching points to compute the fundamental matrix *F*. However, the problem of finding control points between data acquired by different sources with non-common or robust enough features is still an unconsidered field.

In some special cases, a scene is considered as a planar scene. Such a case may possibly be produced when the images baseline is null or the depth relief of the scene is small compared with the extent of the image. In both cases, epipolar geometry is not defined because the epipoles are not accessible and the plane projective transformation is the exact solution to transfer points from one view to another. However, this solution should not be taken as a general rule, because most of the scenes in the man-made environments usually comprise several planes.

On the other hand, it has been demonstrated that the homography induced by a plane$\;\;\pi ={\left(v^{T},d\right)}^{T}$ is determined uniquely by the plane and vice versa, only if the plane does not contain any of the cameras’ centres; otherwise, the homography is degenerated. Suppose the system is a sensory rig set-up; then, the homography matrix is [21]:

(1) $$H=K\prime \left(R-tv^{T}/d\right)K^{-1}$$

The homography matrix is defined by the camera internal (*K*) and external parameters ([*R, t*]) and the plane$\;\;\pi ={\left(v^{T},d\right)}^{T}$. Since the camera parameters are constant, the result in Equation (1) also shows that a family of homographies is parametrized by v/d, where d/‖v‖ is the distance of the plane from the origin. 

Let us assume that a 3D scene reinterpretation is possible by discretizing the scene into *n*-planes. Then, it is also possible to compute *n*-homographies, and transfer the image points from the first view to image points of the second view. Taking advantage of the depth information provided by the ToF camera it would also be possible to compute the approximation of the object planes of the scene. However, such approximation should not be done lightly, because some planes may generate a virtual parallax. Let us suppose that a scene contains two objects; one is represented by a plane angled to the cameras’ views, and the other by a plane in front and parallel to the cameras. Then, the homography induced by the second object (in the front plane) maps incorrectly the points off this plane, in this case the first object. However, if the intersection of these two planes is in the cameras’ views, the points of the intersected line could be properly mapped. Now, instead of using the homography of the first plane (angled object) to transfer it, let us suppose that this angled object is virtually intersected by *m*-planes, all positioned at different distances in front and parallel to the cameras view. Then, there are *m*-lines as a result of these intersections. These *m*-lines describe a discrete shape of the object. Hence, each homography induced by these virtual *m*-planes is able to map its corresponding intersection (*m*-line) on the angled object. This assumption implies that objects into a scene could be explained with a family of virtual *m*-planes, and their induced *m*-homographies are able to map the discrete object’s shape. This homography family only depends on the planes parameters and the distance of the planes to the cameras, similar to Equation (1). However, in this case, the planes do not directly represent the planes on the scenes; they are virtual planes, positioned in front and parallel to the sensory system.

This paper presents a new approach for combining data from ToF and RGB cameras. The method proposes a 3D-space parametrization by virtually discretizing the workspace into *n*-planes. These planes are sequential planes in front of the cameras plane and approximately parallel to the image view. This procedure generates a depth-dependent homography matrices lookup table (*Hlut*). The homography LUT is used for transforming the image points on a 3D differential section (a plane approximation) from the ToF frame to the RGB frame. This transformation also generates a labelled image on the RGB frame. This labelled image corresponds to the indexes *i* of homographies $H_{i}$ used to transform the data. Because of the low resolution of the ToF camera, several adjacent pixels on the RGB image are unmatched. Therefore, in this case a nearest-neighbour-based algorithm is adopted on the labelled image, and transformation matrix$\;H_{i}$ is assigned to the unmatched data on the RGB image. Now, the transformation from the RGB to the ToF frame is the straightforward computation of the high resolution RGB-D map. This research has considered a depth of field of the workspace from 300 mm to 1300 mm. The proposed solution is intended to be used in any dynamically changing environment under near-real-time conditions, and without any previous knowledge of the scene. 

## 2. Materials and Methods

This section describes the sensory system utilized for the data acquisition and explains the proposed methodology for combining the ToF and the RGB cameras and computing high resolution colour depth maps.

### 2.1. Sensory System Configuration 

**Figure 1:**
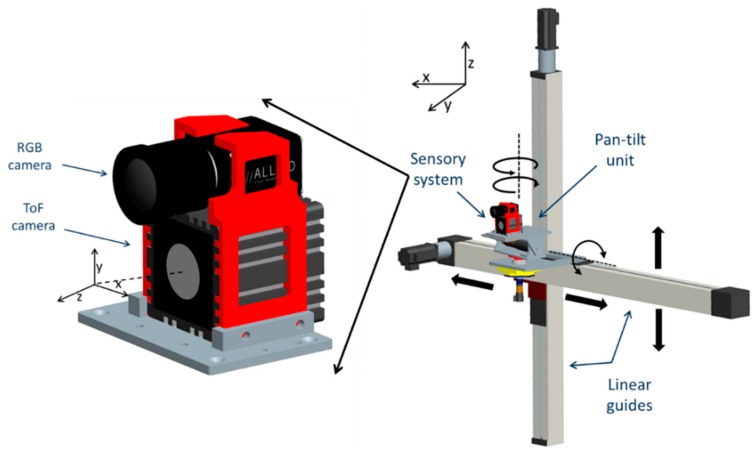
Sensory system configuration. The sensory rig consists of a ToF camera and a RGB camera, and it is mounted on a robotic platform with four degrees of freedom.

The sensory system consists of a high resolution colour camera and a 3D ToF camera. The ToF camera of the system is the SR4 Mesa SwissRanger [6] with a resolution of 176 × 144 pixels and a frame rate of 30 fps. The ToF camera provides three images: the amplitude response, the confidence map and the depth map. The depth map could also be converted to xyz Cartesian coordinate data, with the origin of the coordinated system in the centre front of the camera, with Z coordinate increasing along the optical axis away from the camera, Y coordinate increasing vertically upwards and X coordinate increasing horizontally to the left (see Figure 1). For the RGB camera, the AVT Prosilica GC 2450 [23] was used. The camera resolution is 2448 × 2050 pixels and its frame rate is up to 15 fps.

The cameras are vertically aligned and placed as close as possible to each other. The sensory system is mounted on a four degrees of freedom robotic platform. This platform consists of two prismatic joints and a pan-tilt unit. The prismatic joints provide the vertical and horizontal movements in the XZ Cartesian plane. The rotational joints on the pan-tilt unit provide the pitch and yaw movements of the system [24]. The joints properties of the platform are described in Table 1. The system configuration is depicted in Figure 1.

**Table 1:** Properties of the robotic platform joints.

Joints/Axis	Max. Velocity	Operating Ranges	Accuracy
Vertical	500 mm/s	±700 mm	±0.1 mm
Horizontal	500 mm/s	±700 mm	±0.1 mm
Pitch	40 rpm	±30°	±0.0012°
Yaw	81 rpm	±360°	±0.0024°

### 2.2. Depth-Dependent Homographies Matrix Lookup Table for Depth Registration

The primary goal of this work is the generation of high resolution colour depth maps under real time conditions by using the data acquired by ToF and RGB cameras. Commonly, registration methods aim the geometrical alignment of two (or more) images of the same scene by means of a feature based method. These images might be acquired by different sensors, from different views or taken at different times. An extensive review is presented by Zitová and Flusser [25]. In this setup configuration, finding robust features between depth maps and colour information is not possible, and only artificial landmarks might be matched properly. Since the method should work under natural conditions, landmarks are not the proper solution. Normally, depth map registration is done by computing the extrinsic parameters of the coordinate transformation between the two cameras. The 3D points from the available depth data are back-projected to the colour image and a low resolution depth colour map is obtained. Some works have been dedicated to increase the resolution of the dense map by upsampling techniques and presenting very interesting results. However, most of them are not suitable for near real time applications. Other methods adopted interpolation algorithms for the depth upsampling before transferring the data, though the maximum dense map size reported is 1.5 Megapixels. Some works have reported difficulties on the objects edges, this problem is mainly produced by false depth values or the over smooth depth value, caused by the data interpolation. 

**Figure 2:**
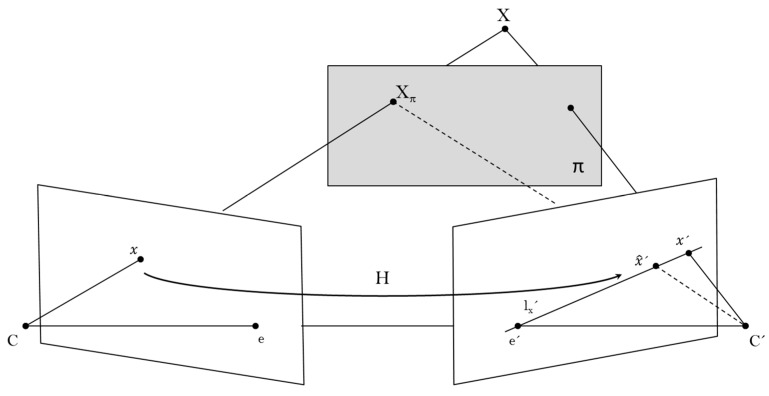
Plane induced parallax.

The proposed approach relies on uncalibrated techniques for transferring points from one view to another. Normally, the epipolar geometry is the features based solution for computing correspondences of 3D-space points between two views, but in similarity with registration methods, matching robust features is also not achievable within this context. On the other hand, planar projective transformation does not require the search of features once the homography is computed. In some cases, the scenes might be considered as planar scenes, but most of natural scenes consist of several planes and the objects into the scene are considered non-planar objects. Now, let us assume that $H_{\pi }$ is the homography induced by the plane π. Then, suppose that when mapping 3D-space points between the two views, some of these points are off the plane π. In such a case, the homography generates a virtual parallax; a schematic illustration is displayed in Figure 2. The 3D point X is off the plane π, thus the ray through X intersects π at some point $\;X_{\pi }$. These two 3D points are coincident in the first view at point x, but in the second view, the images of X and $\;X_{\pi }$ are not coincident. The vector between ${\hat{x}}^{\prime }$ and x′ is the parallax relative to$\;H_{\pi }$.

**Figure 3:**
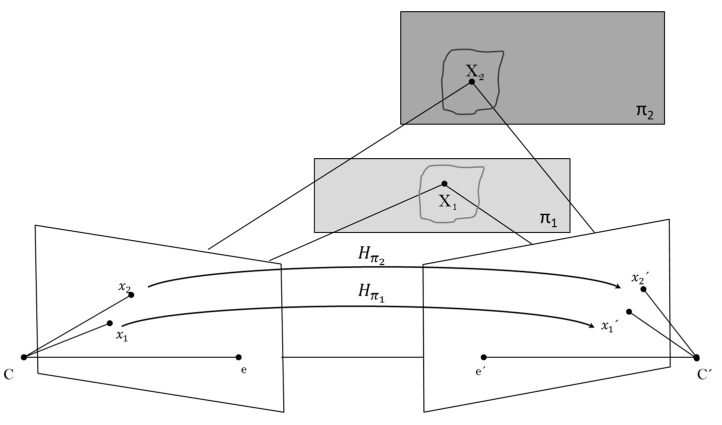
Plane projective transformation induced by two planes $\pi_{1}$ and$\;\pi_{2}$ on a scene.

Assume from the scene above that the two points $X_{1}=X_{\pi }$ and$\;X_{2}=X$ are on plane $\pi_{1}$ and$\;\pi_{2}$, respectively, and $H_{\pi_{1}}$ and$\;H_{\pi_{2}}$ are homographies induced by the corresponding planes. If the ray through each 3D-space point is not coincident neither in the first view nor in the second view, then the images of the points are $x\prime_{1}=H_{\pi_{1}}x_{1}$ and$\;x\prime_{2}=H_{\pi_{2}}x_{2}$ (see Figure 3).

Along this idea, suppose that scenes composed by *n*-objects could be approximated to *n*-planes and consequently, *n*-homographies could be computed. This assumption should be prudently considered, because objects with large relief or positioned closed to the sensory system, certainly are explained with more than one plane. Under these circumstances, a unique homography approximation of an object also generates a virtual parallax. In this case, let assume the object is virtually intersected by *m*-planes, all positioned in front and parallel to the cameras. Then, each of these intersections generates *m*-silhouettes of the object shape. Hence, each homography induced by these virtual *m*-planes is able to map its corresponding intersection, the *m*-silhouettes of the object. 

**Figure 4:**
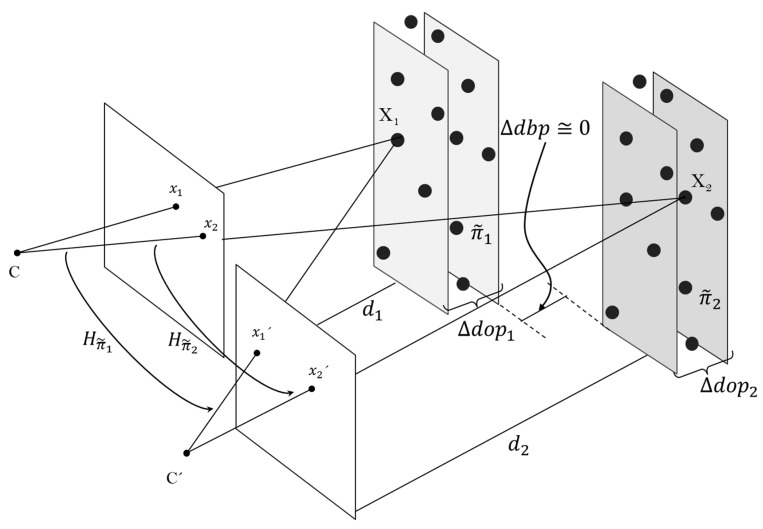
Formation of the depth-dependent homography lookup table.

The approach of this work proposes an alternative 3D world parametrization by virtually discretizing it into *n*-planes and thus, computing a depth-dependent homography lookup table. These *n-*planes are parallel to the sensory system and sequentially positioned in front of it. Taking advantage of the depth information available from the ToF camera, the distance of each *n*-plane from the camera is known. A 3D-space plane (${\tilde{\pi }}_{i}$) in the discretizing process is represented as a volumetric unit. This unit is composed by 3D points hold within a depth differential section, denoted as differential of depth of a plane (∆dop). The dimension of $∆dop_{i}$ is directly proportional to the distance from the plane ${\tilde{\;\pi }}_{i}$ to the sensory system. For instance, the closer the object is to the vision system, the larger its relief is compared with the extent of the image, and the higher the number of *n-*planes is for explaining the object and the smaller is the $∆dop_{i}$. Henceforth, the matching feature for the image registration method is the distance from 3D-space points to the ToF camera ($d_{i}$). A plane ${\tilde{\pi }}_{i}$ is approximated from a cluster of 3D points if and only if, its induced homography maps their images points from one view to another within errors less than 3 pixels on the RGB frame. The distance between planes (∆dbp) should be approximately equal to zero. Figure 4 shows an illustration of the discretizing process and the depth-dependent homography lookup table formation.

For computing the depth-dependent *Hlut*, 104 images of a known pattern grid were captured. In order to avoid unreliable depth measures because of dark objects, the pattern grid is a 3 × 5 white-red chessboard of 50 mm each square. The effective pattern is the inner 2 × 3 grid, thus, the 12 control points on the board $\left\{X_{i}\right\}$ are 12 image correspondence points on each view $\left\{xg_{j}^{ToF}\right\}↔\;\left\{xg_{j}^{RGB}\right\};\;$ j=1…M, M=12. Then $\left\{\;xcp_{i}^{ToF}\right\}\;and\;\left\{\;xcp_{i}^{RGB}\right\};i=1\ldots N,N=104$ are *N* samples of the 12 grid points, where $xcp_{i}^{ToF}\;∋\left\{xg_{j}^{ToF}\right\}\;and\;\;\;xcp_{i}^{RGB}\;∋\left\{xg_{j}^{RGB}\right\}$. These points are extracted from RGB images and grayscale amplitude images, these last ones provided by the ToF camera. From this point forward, when referring to ground control points, it is assumed that it is referred to $\;xcp_{i}^{ToF}\;and\;\;\;xcp_{i}^{RGB}$.

**Figure 5:**
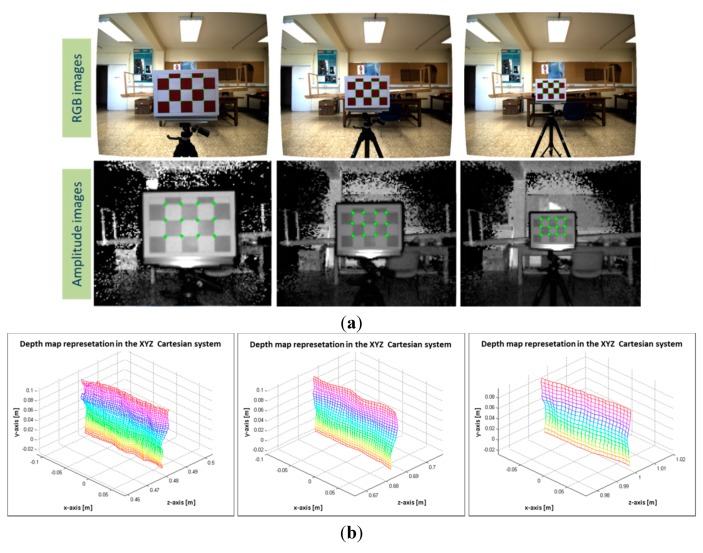
Samples of images pair of the pattern grid board. (**a**) RGB and ToF amplitude images; (**b**) the depth map representation in the Cartesian system of the inner 2 × 3 grid.

The board was positioned at several distances in front of the sensory system and approximately parallel to it. The 104 image samples are different poses of the pattern board, where the pattern was sequentially positioned at distances from 300 mm to 1300 mm from the board to the sensory system. The distance from the pattern to the sensory system is calculated by using the depth information enclosed in the inner 2 × 3 grid. This region is extracted for computing the mean depth and subsequently, the distances $\;d_{i}$ from the board to the system. An example of image pairs from the RGB image and the ToF amplitude, and their ground control correspondence points are shown in Figure 5a. The 3D view of the region enclosed in the inner grid of the board is displayed in Figure 5b. Notice that both images and the depth information have been previously undistorted before extracting the ground control points.

The homography computation was carried out by following the normalized direct linear transform (DLT) algorithm and the RANSAC method for robust model approximation in presence of outliers [21[26]. The initial step was the calculation of sets of homographies by gathering combinations of ground control points of the *N* image sample $\;\left\{\;xcp_{i}^{ToF}\right\}\;and\;\left\{\;xcp_{i}^{RGB}\right\};i=1\ldots N,N=104$. Only homographies capable of mapping points within absolute geometric error per point less than 2 pixels on any image axis (*u, v*) are selected. However, in order to avoid error misleading because of the outliers on the selection of the grid control points, the overall absolute error is used. The overall error is computed with the sum of the error points, and since the number of grid points is 12 and the points has 2 image axes (*u, v*), the maximum overall error is 48. This error is measured when mapping points from the ToF to the RGB frame and it is the absolute difference between the estimated points and the ground control points. The sets of points mapped are denoted as $\;\left\{\;xmap_{i}^{ToF}\right\}\;and\;\left\{\;xmap_{i}^{RGB}\right\}$ and the grid points as$\;\left\{\;xgm_{i}^{ToF}\right\}\;and\;\left\{\;xgm_{i}^{RGB}\right\}$ respectively, so the absolute error of the estimated sample is:

(2) $$\epsilon =\left|xcp_{i}^{RGB}-\;xmap_{i}^{RGB}\right|\overset{overall}{\Rightarrow }\overset{12}{\underset{j=1}{\sum }}\left|xg_{j}^{RGB}-xgm_{j}^{RGB}\right|<48$$

The next step is to remove the duplicated homographies, which involves the utilization of the transformation matrices computed by the same combination of control points $\;\left\{\;xcp_{i}^{ToF/RGB}\right\}$. At this stage, a list of potential homographies is achieved. Each of them is related to a list of its properly mapped samples $\;\left\{\;xcp_{i}^{ToF/RGB}\underset{H}{\to }xmap_{i}^{ToF/RGB}\right\}$. Since the distance $d_{i}$ from the board to the sensory system at any sample is known, a list of minimum and maximum working distances related to each homography is created as well$\;\left(dmin_{i},dmax_{i}\right)$.

The final step of the procedure is the selection of the optimal entries for the homography lookup table such that $\;Hlut\;∋\;H_{i}^{lut},dmin_{i}^{lut},dmax_{i}^{lut}\;\;i=1\cdots numH$. For that purpose, some conditions should be satisfied. The *Hlut* should cover the entire depth of field [300–1300 mm] of the parametrized 3D world. The distance between homographies (∆dbp≅0) should be approximately equal to zero, and the number of entries on the LUT should be as minimal as possible. Algorithm 1 shows the pseudocode for computing the depth-dependent *Hlut* by using the ToF and the RGB cameras.

**Algorithm 1** Automatic estimation of the depth-dependent homography lookup tableObjective: given
*N* samples of sets of 2D to 2D correspondence points$\;\left\{\;x_{i}\right\}↔\left\{x\prime_{i}\right\}$, compute a depth-dependent homography lookup table $Hlut=\left\{H_{k}^{lut}\right\}$ such that $x\prime_{i}=H_{k}^{lut}x_{i}$. These sets are the projected image points of the 3D-space points$\;X_{i}$, which are distributed at several distances from the system, and parallel to it.1. Acquire ToF and RGB images pairs of
*N* different poses of a known pattern, where numSamples={1⋯N}. The pattern is a white-red chessboard sequentially positioned in front and approximately parallel to the sensory system.2. Extract the
*M* grid correspondence points of each image sample from the previous step (*i*) to compose the *N* sets$\;x_{i}↔x\prime_{i}$.3. Apply the DLT algorithm to compute homographies by combining sets of the 2D to 2D correspondence points such that
$\;xx\prime_{j}=H_{k}xx_{j}$, where$x_{a}\cup^{\text{​}}\cdots \cup^{\text{​}}\;x_{n\;}=\left\{xx_{j}:xx_{j}\;\in x_{g}\;where\;a\leq g\leq n\;and\;a,n\;\in numSamples\right\}\;$and$\;\;x\prime_{a}\cup^{\text{​}}\cdots \cup^{\text{​}}\;x\prime_{n\;}=\left\{xx\prime_{j}:xx\prime_{j}\;\in x\prime_{g}\;where\;a\leq g\leq n\;and\;a,n\;\in numSamples\right\}$.4. Compute the absolute geometric error between the mapped points
$\;{\hat{x}}_{i},\;\hat{x}\prime_{i}$ and the measured point$\;\;x_{i},\;x\prime_{i}$ such that$\;\;\epsilon ={\left|x_{i}-\hat{x}\right|}_{i}$ and$\;\;\epsilon \prime ={\left|x\prime_{i}-\hat{x}\prime \right|}_{i}$.5. Create a list of homographies that map points within error less than 3 pixels on the highest resolution image frame.6. Remove duplicated homographies and define a list of potential homographies
$\;H_{k}$.7. For each element of the list in (
*vi*), compute the maximum and minimum working distance from the depth information of the set of 2D-2D correspondence points of (*iii*), such that $dmax=\underset{a\leq g\leq n}{\max}dg,\;dg=\left\{d_{a}\cup^{\text{​}}\cdots \cup^{\text{​}}d_{n}\right\};a,\;n\in numSamples$ and$\;dmin=\underset{a\leq g\leq n}{\min}dg,\;d_{g}=\left\{d_{a}\cup^{\text{​}}\cdots \cup^{\text{​}}d_{n}\right\};\;a,n\;\in numSamples$.8. Select the optimal transformation matrices to create the depth-dependent homography lookup table where
$Hlut\;∋\;H_{i}^{lut},\;dmin_{i}^{lut},\;\;dmax_{i}^{lut}\;\;i=1\cdots numH$. For that:
Limit the depth of field by$\;dof^{hlut}=\left[dmin_{1}^{lut},dmax_{numH}^{lut}\right]$.Approximate the distance between homographies to zero$\;∆dbp_{i}^{lut}≅0$, where$\;∆dbp_{i}^{lut}=dmin_{i+1}^{lut}-dmax_{i}^{lut}$.Minimize the elements of the lookup table$\underset{a\leq numH\leq n}{\text{ min}}numH$.

### 2.3. Validation of the Depth-Dependent Homography Matrices Lookup Table

The transformation from the ToF to the RGB frame was considered for the method evaluation. Since the method is depth-feature-based, the procedure input is the depth information provided by the ToF camera. The uncertainty because of the difference between the cameras resolution is a crucial issue for evaluating the proposed registration method. For any ToF point there are several potential correspondence points on the RGB frame. By this means, the geometric error in (*uv*) axis of the 12 ground control points from the 104 image samples was analysed. The pseudocode for mapping points from the ToF to the RGB frame by using the depth-dependent *Hlut* method is presented in Algorithm 2. 

**Algorithm 2** Procedure for mapping points between two views (ToF → RGB) based on the depth-dependent *Hlut*.1. Extract the mean depth of ROI of the control points
$\;d_{i}^{pt}$.2. Find the corresponding entry
*k* on the *Hlut* that suits$\;d_{i}^{pt}$ such that$\;\;dmin_{k}^{lut}\leq d_{i}^{pt}\leq \;dmin_{k}^{lut}$.3. Compute the transformation of the points by applying the homography
$\;H_{k}^{lut}$ such that$\;xmap_{i}^{RGB}=\;H_{k}^{lut}\;xcp_{i}^{ToF}$.

**Figure 6:**
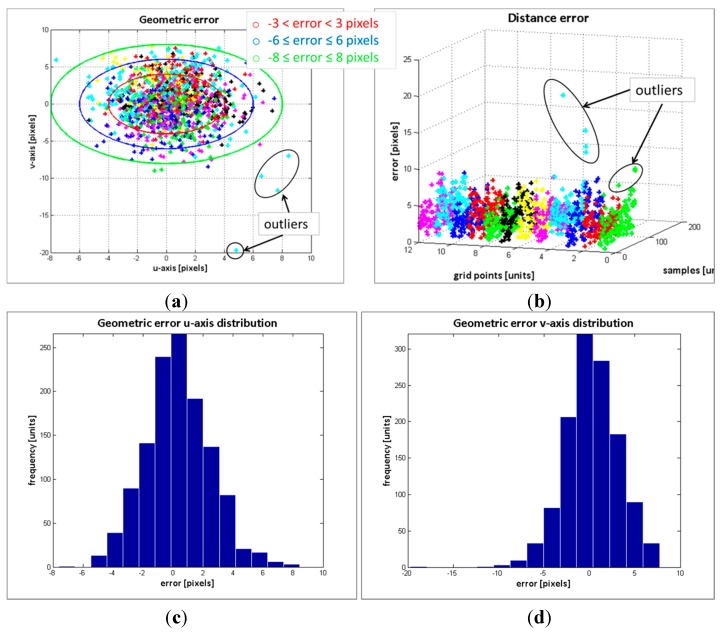
Geometric error evaluation. (**a**) geometric error; (**b**) distance error; (**c**) error distribution in *u*-axis; (**d**) error distribution in *v*-axis.

The result of the geometric error of the control points from the 104 image samples is depicted in Figure 6a. In Figure 6b, the distance error of the points with respect to their position on the pattern board and image sample is displayed, while the distribution of the error on the *u*-axis and *v*-axis are in Figures 6c and d, respectively. Table 2 summarizes the results of the error distribution.

**Table 2:** Results of the error distribution.

Error Distribution (pixels)	Error Percentage (%)	
	*u-Axis*	*v-Axis*
error≤|3|	82.9	70.22
|3|<error ≤ |6|	16.2	25.2
|6|<error ≤ |8|	0.8	4.1
error> |8|	0.1	0.48

The results in Figure 6 along with the data in the Table 2 indicate that the error deviation in the *v*-axis is higher than the error in *u*-axis. Since the cameras are vertically aligned, such behaviour was expected. Regarding the errors distribution, the standard deviation of the error in the *v*-axis is  σ=3.19, and at least the 70.22% of the estimated data has an absolute error less or equal than 3 pixels (see Figure 6d and Table 2). Only the 4.58% of the data has an absolute error higher than 6 pixels. The maximum absolute error is 20 pixels. For the error distribution in the *u*-axis, the maximum error is ±8 pixels and the standard deviation is σ=2.78. Most of the absolute error is less or equal than 3 pixels, exactly the 82.9% of the data, and only the 0.9% of the absolute error is higher than 6 pixels. 

In general, when analysing the error within the RGB frame (2448 × 2050 pixels), the relative errors are significantly low. An error of 3 pixels represents a relative error of 0.15% over the RGB image, and for the 20 pixels deviation, a relative error of 0.9% is reached. Though these values are evidently small, it is still something to be concerned with. Several conditions might introduce error to the method, for instance:

1. The outliers in the selection process of the correspondence control points;
2. The presence of noise in the depth measurements;
3. The implicit error of the transformation matrices.

**Figure 7:**
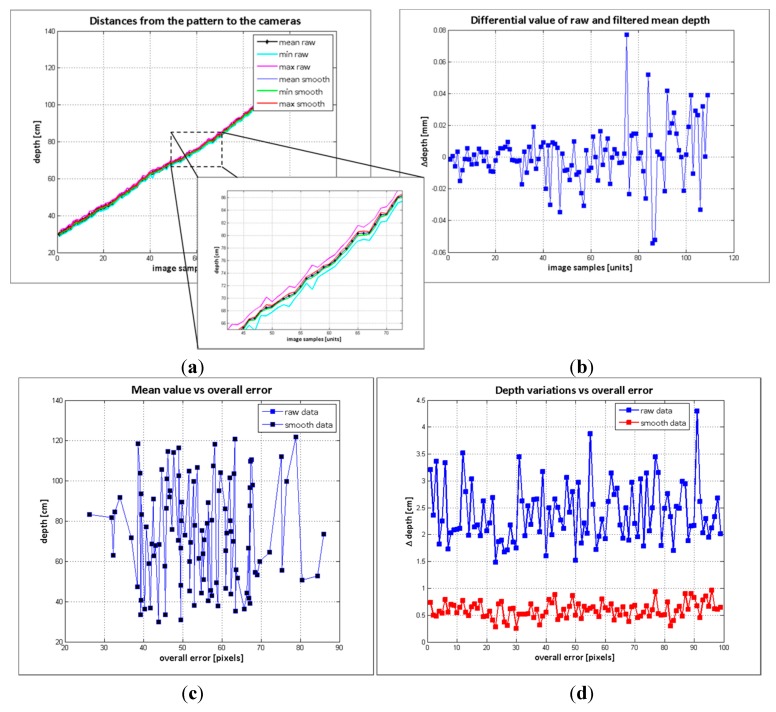
Depth measurements evaluation. (**a**) distances from the pattern board to the cameras; (**b**) differential value of raw and filtered data; (**c**) samples mean depth *vs*. overall error; (**d**) samples maximum variation vs overall error.

In order to evaluate the influence of the depth variations on the proposed method, the depth measurements of the effective grid pattern were analysed. Two groups of data were compared: the raw depth and the filtered depth. For smoothing the depth data, the denoising algorithm proposed in [27] was adopted, and Figure 7 shows the results of the analysis. In Figure 7a the mean depth and the depth boundaries of the pattern board acquired in the 104 image samples are shown. The raw depth has higher data variance, though the mean of the raw and filtered data are nearly the same, as it is illustrated in Figure 7b. The impact in the overall error because of the object distance and the object depth variations are illustrated in Figure 7c and d. According to these results, neither the mean distance nor the maximum depth variations have direct correlation to the error’s scope. Therefore, it is possible to conclude that the error is not reliant on the depth variations within 25 mm, corresponding to the mean maximum depth variations of the raw data. 

**Figure 8:**
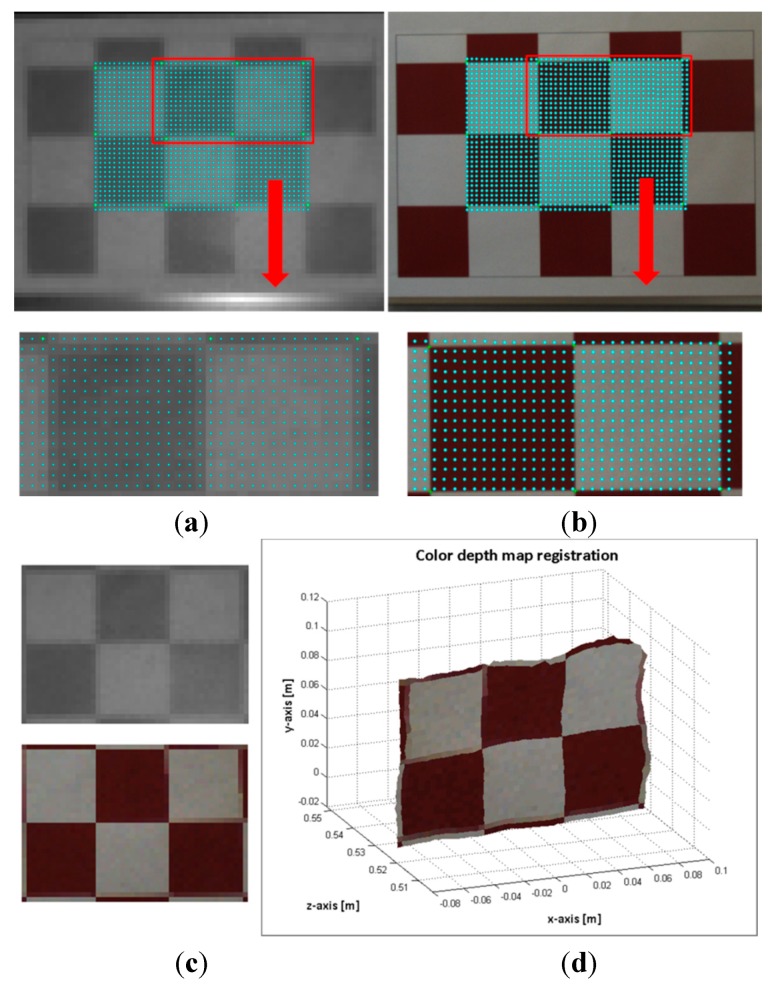
Image sample 49-pattern board positioned at 527 mm. (**a**) Top: selected ROI on the ToF image. Bottom: zoom of the selected points on the ToF image; (**b**) Top: mapped points on the RGB image. Bottom: zoom of the estimated points on the RGB image; (**c**) Top: ROI of the ToF image. Bottom: Composition ROI from the mapped points on the RGB image; (**d**) colour depth map.

**Figure 9:**
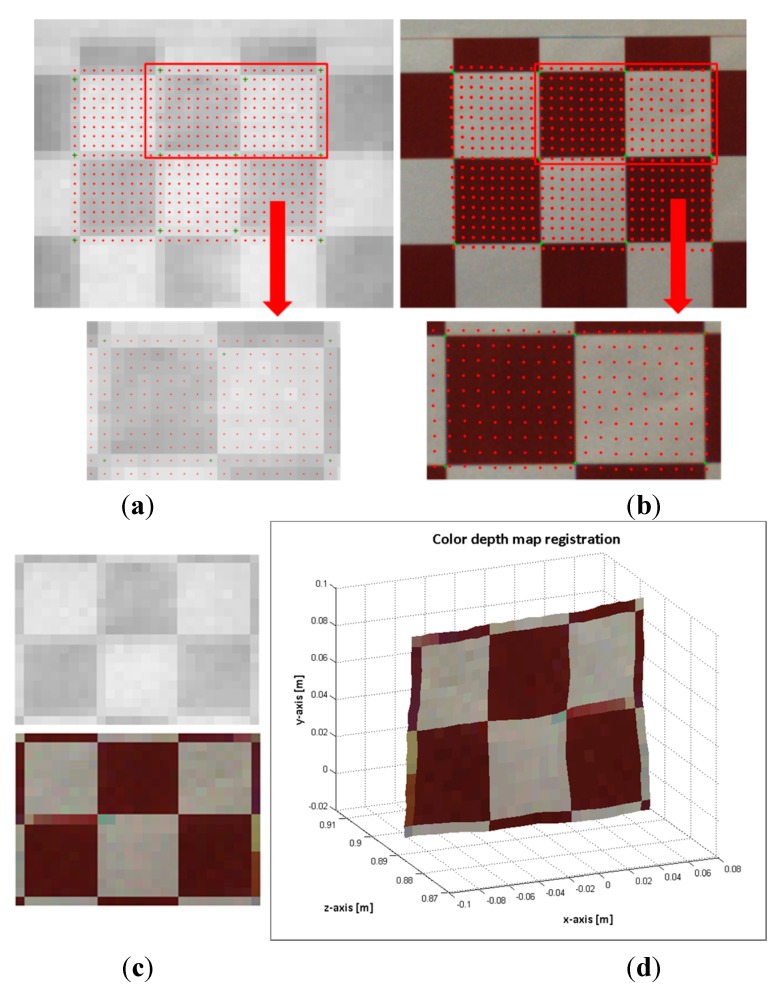
Image sample 25-pattern board positioned at 527 mm. (**a**) Top: selected ROI on the ToF image. Bottom: zoom of the selected points on the ToF image; (**b**) Top: mapped points on the RGB image. Bottom: zoom of the estimated points on the RGB image; (**c**) Top: ROI of the ToF image. Bottom: Composition ROI from the mapped points on the RGB image; (**d**) colour depth map.

The flawed points selected as correspondence control points could be the most frequent problem for introducing error on the estimated data. Consequently, instead of a point to point evaluation, entire regions of the images with higher errors were analysed. Figure 8 and Figure 9 shows the evaluation results of two images of the pattern board; in one image the board is positioned at 527 mm and in the other at 891 mm. First, a region of interest (ROI) in the ToF image is selected. Then, the depth measures of the ROI are sorted in ascending order, and clusters of 12 mm of standard deviation are created$\;c^{j}=\left\{x_{i}^{ToF}\right\}$. Finally, the mean depth $\;dm_{j}^{c}$ of each of these clusters $\left(\;c^{j}\right)$ is matched with a suitable distance entry *k* on the *Hlut*, such that$\;\;dmin_{k}^{lut}\leq dm_{j}^{c}\leq \;dmax_{k}^{lut}$. Thus a homography $\;H_{k}^{lut}$is designated to each $\;c^{j}$, and the selected ROI is mapped as$\;\forall \;c^{j}:\;\left\{xmap_{i}^{RGB}\right\}=H_{k}^{lut}\left\{x_{i}^{ToF}\right\}$. In the images displayed in Figure 8a,b and Figure 9a,b, the mapped points are marked with dots. The colour of the dots indicates the entry *k* of the homographies $\;H_{k}^{lut}$ used to transfer the data. The composition of the ROI from the estimated points on the RGB image and the ROI of the selected points on the ToF are illustrated in Figure 8c,d and Figure 9c,d.

Since the RGB and ToF images are acquired from distinct sources and there is a large difference between their image resolutions, the properties of the sensed objects tend to be different. The most relevant effects are perceived in the borders of the textured objects and in the objects dimensions. Let us utilise the image capturing of the pattern board by way of illustration (see Figure 8 and Figure 9). The RGB high resolution camera acquisition sharpens the squares borders, while the capturing by the low resolution ToF camera unsharpens the squares borders of the pattern. Thus, the squares on the composed ROI from the mapped points on the RGB image are slightly blurred. Additionally, a great number of elements can affect the response of the ToF camera, for instance the rays emitted from the sensor that lie on the object’s edge tend to be less accurate because they are affected by the multi-path interferences. Consequently, the objects dimensions on the ToF image are not always alike compared with the ones on the RGB image. In Figure 8, some of these issues are illustrated. As an example, the pattern board is smoothly rotated with respect to the optical axis of the cameras; this rotation is only perceived by the RGB camera. 

Regarding the large difference in the cameras resolution, there are several unmatched points (~10–11 pixels) between adjacent estimated points on the RGB image. Nonetheless, the results of the proposed method shows that the region composed of the estimated points on the RGB image and the selected region on the ToF image are very close to each other, and proportionally registered.

### 2.4. Comparison with the Standard Calibration Method (Homogenous Transformation)

The methods comparison was focused on the evaluation of their response to the noise in the depth estimations. The noise in the measurements is a persistent problem; usually it is produced as a function of the objects albedo and the depth discontinuities on the objects edges. For that matter, a white-black chessboard was chosen as a target for reproducing noisy depth measurements. First, the standard calibration of the sensory system was carried out, where 62 images samples of a chessboard were acquired and the Matlab Camera Calibration Toolbox [28] was used to estimate the calibration parameters. For acquiring these 62 image pairs, composed by an RGB image and a ToF amplitude image, the pattern board was located at different poses and orientations, and at different distances from the sensory system. Then, 30 correspondence ground truth points were selected on each of the 62 image pairs. For evaluating the results of the standard calibration parameters, the control points on the amplitude images were transformed to the world coordinate and then, these 3D points were back-projected to the RGB image. The obtained results, with an  RMSE=0.0667, show that the computed calibration parameters for the Homogenous Transformation provide accurate data fusion. 

In order to compare the results of the standard calibration method with the results of the depth-dependent *Hlut* method for the depth map registration, the real raw depth measurements of the pattern board of the 62 image samples were used. The data fusion by means of the standard calibration technique was carried out by implementing the method described in [14], were the depth measurements are used to back-project the 3D world points to the 2D points on the RGB image. In the case of the depth-dependent *Hlut* approach, the depth map registration was done by following the procedure described in Algorithm 2 (see Section 2.3).

Regarding the numerical results of the error evaluation of the 62 image samples, in terms of RSME, the proposed method of this work reduces the error in 25% in comparison with the standard calibration method. The RMSE for the standard calibration method is 0.6517 and for the proposed method is 0.4892. Thus, the error evaluation of both methods indicated that the depth-dependent *Hlut* approach outperforms the standard calibration method, when using raw (unfiltered) depth measurements from the ToF camera. In Table 3, more information regarding the errors distribution is shown. These results also show that the proposal of this work has better response to noise in the depth measurements.

**Table 3:** Results of the error distribution.

Error Distribution (pixels)	Error Percentage (%)					
	*Standard Calibration Parameters*				*Proposed Method (Hlut)*	
	*Calibration Grid Data*		*Depth Values*			
	*u-Axis*	*v-Axis*	*u-Axis*	*v-Axis*	*u-Axis*	*v-Axis*
error≤|6|	99.99	100	20.7	42.2	40.6	65.9
|6|<error ≤ |10|	0.01	0	16.2	23.6	37.7	19.1
|10|<error ≤ |14|	0	0	13.2	17.7	13.8	6.8
error> |14|	0	0	49.9	16.5	7.9	8.2

### 2.5. High Resolution Colour Depth Map Estimation

The approach for computing the high resolution colour depth map is presented in Algorithm 3. The procedure is based on the depth-dependent *Hlut* and a nearest neighbour classification algorithm. This section is focused on pointing out the potential of the registration method proposed in this paper. In future investigations, more robust and complex classification methods could be adopted for increasing the method precision and the quality of the high resolution colour dense map.

**Algorithm 3** Procedure for mapping points between two views (ToF ↔ RGB) based on the depth-dependent.1. Select a ROI in the ToF image for data registration or select the entire image.2. Sort in ascending order the depth measures of the selected region and create
*Q* clusters with 12 mm of standard deviation such that$\;c^{j}=\left\{x_{i}^{ToF}\right\},\;j=1\cdots Q$ and compute the mean depth of each cluster$\;dm_{j}^{c}$.3. Find the corresponding distance entry
*k* on the *Hlut* that suits the mean depth of each cluster, such that$\;\forall \;c^{j}:\;\left\{xmap_{i}^{RGB}\right\}=H_{k}^{lut}\left\{x_{i}^{ToF}\right\}\;|\;\;dmin_{k}^{lut}\leq dm_{j}^{c}\leq \;dmax_{k}^{lut}$, where j=1⋯Q and1≤k≤numH.4. Compute the points transformation from the ToF to the RGB images by using the homographies
$\;\left\{\;H_{k}^{lut}\right\}$ designated in the previous step.5. Create a labelled image (LRGB) corresponding to the RGB frame, where the values for the mapped points are the
*k* entries of$\;\left\{\;H_{k}^{lut}\right\},\;1\leq k\leq numH$, and the values for the unmatched points are zero.6. Approximate the unmatched pixels of the labelled image (LRGB) by applying the nearest neighbour classification algorithm.7. Compute points transformation from RGB to ToF images such that
$\;xmap_{i}^{ToF}=H_{k}^{{lut}^{-1}}x_{i}^{RGB}$.

**Figure 10:**
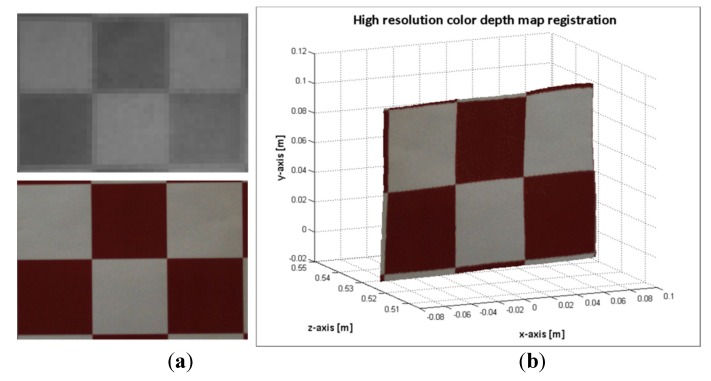

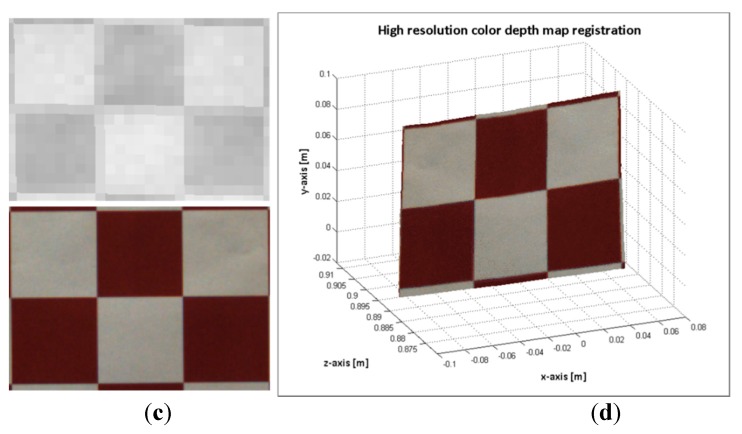
Image sample 49. (**a**) Top: mapped points on the ToF image. Bottom: points of the ROI on the RGB image; (**b**) high resolution colour depth map; Image sample 25; (**c**) Top: mapped points on the ToF image. Bottom: points of the ROI on the RGB image; (**d**) high resolution colour depth map.

In Figure 10, the results of computing a high resolution colour dense map by using the procedure listed in Algorithm 3 are shown. The procedure was applied to images 25 and 49; the same images are presented in section 2.3 (see Figure 8 and Figure 9). 

## 3. Experimental Section

**Figure 11:**
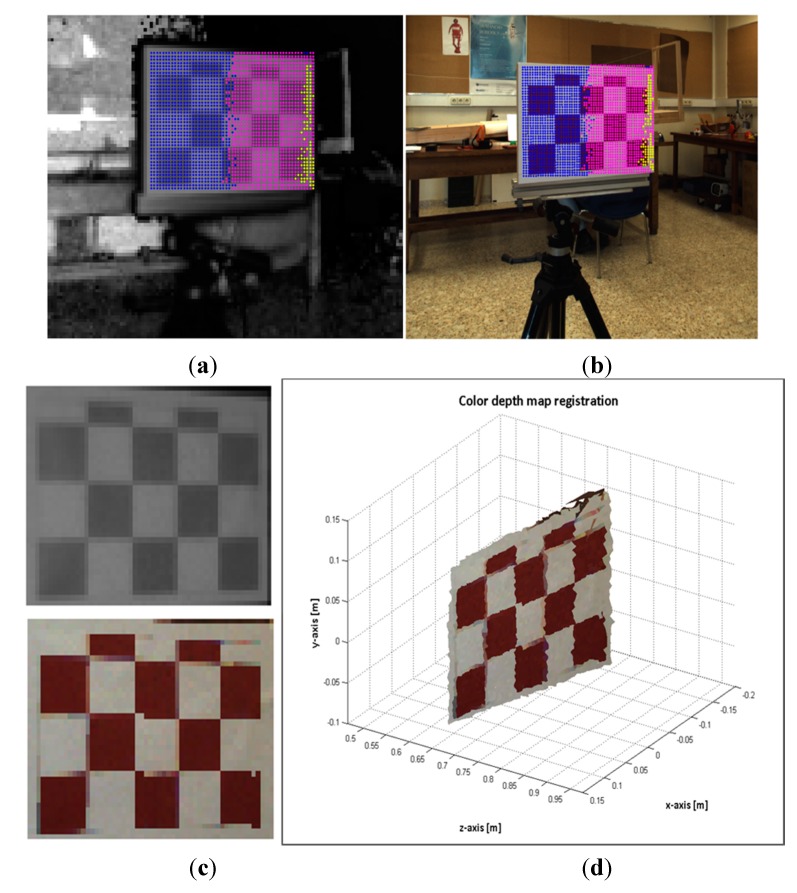
Image sample 19. (**a**) selected points on the ToF; (**b**) estimated points on the RGB image; (**c**) Top: selected ToF ROI. Bottom: estimated RGB ROI; (**d**) colour depth map of the ROI.

**Figure 12:**
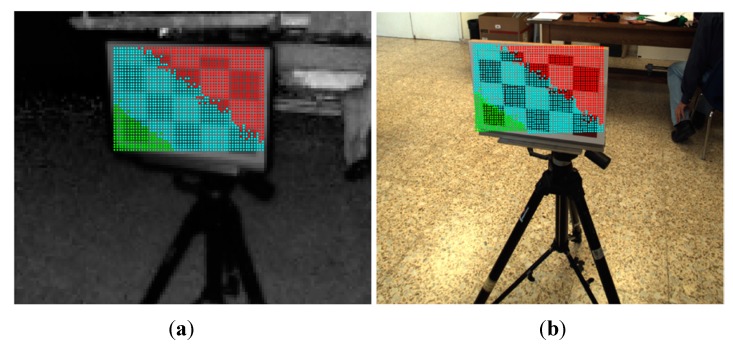

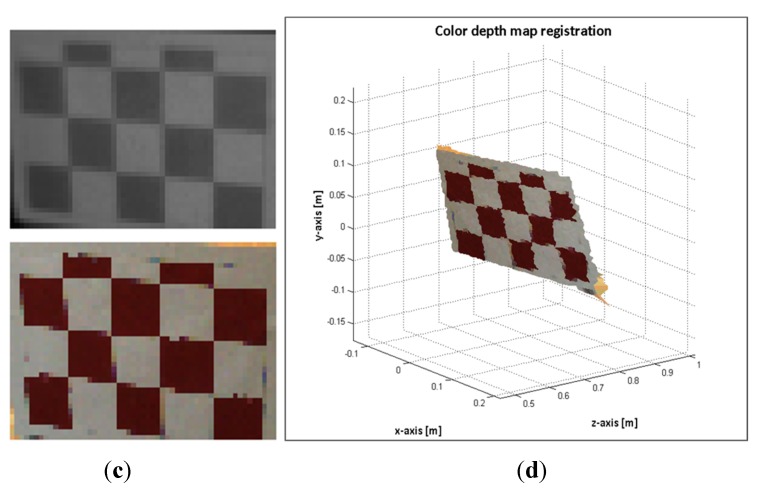
Image sample 25. (**a**) selected points on the ToF; (**b**) estimated points on the RGB image; (**c**) Top: selected ToF ROI. Bottom: estimated RGB ROI; (**d**) colour depth map of the ROI.

In order to evaluate the registration method proposed in this work, two series of experimental tests were conducted. The first group was focused on registering planar object from different perspectives with respect to the sensory system. Thus this means an angled continuous planar surface might be transformed by several homographies $\;\left\{\;H_{k}^{lut}\right\}$. This was achieved by modifying the perspective view of the white-red pattern board. The experimentation setup consisted of the four degrees of freedom robotic platform, the 3 × 5 white-red chessboards of 50 mm each square and the sensory system. The sensory system was mounted on the robotic platform (see Section 2.1 for the platform description). The pattern board was positioned in front of the cameras and the inner grid was aligned with the centre of the ToF camera. Then, 25 images from different poses of the sensory rig were acquired. Additionally, another 37 images of the pattern at different positions and distances were also captured. The results of the registration procedure of two image samples are illustrated in Figure 11 and Figure 12. The transformation matrices $\;\left\{\;H_{k}^{lut}\right\}$ applied for transferring the points are represented with coloured marks; each colour represents an entry *k* of each homography$\;\left\{\;H_{k}^{lut}\right\}$. 

**Figure 13:**
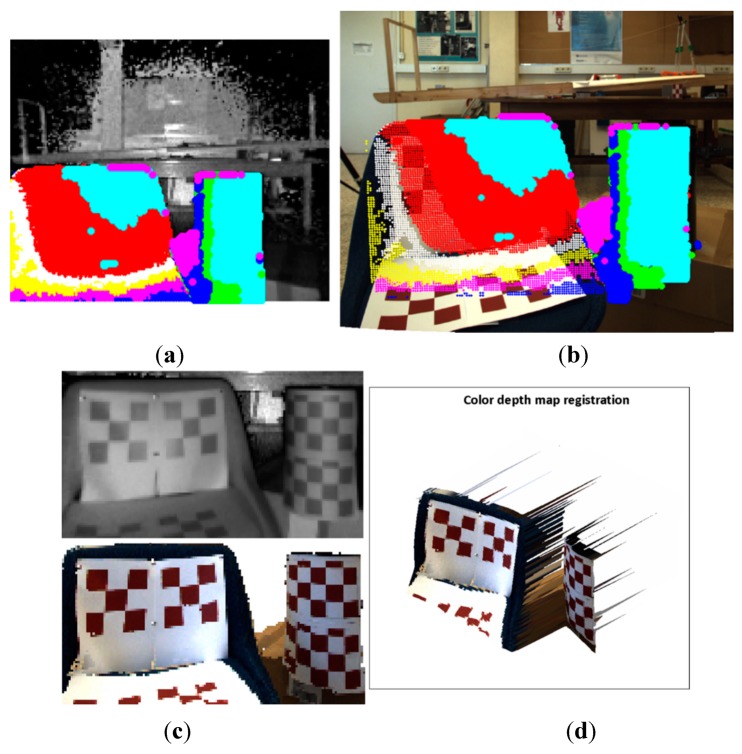
Image sample 22. (**a**) Selected points on the ToF; (**b**) estimated points on the RGB image; (**c**) Top: selected ToF ROI. Bottom: estimated RGB ROI; (**d**) the colour depth map of the ROI.

**Figure 14:**
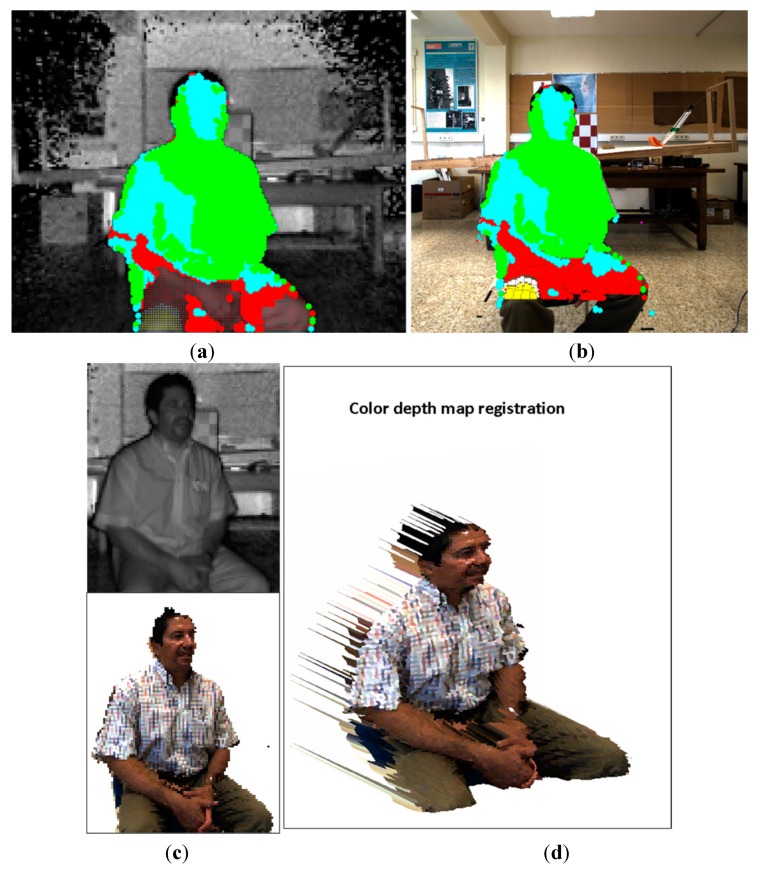
Image sample 33. (**a**) Selected points on the ToF; (**b**) estimated points on the RGB image; (**c**) Top: selected ToF ROI. Bottom: estimated RGB ROI; (**d**) the colour depth map of the ROI.

The second group of experiments are intended to evaluate the proposed method for registering images of 3D man-made scenes. These scenes are composed by volumetric objects made of different materials, and it is expected that the relief of the 3D objects is high enough with respect to the extent of the image view. Therefore, 3D-space points of an object do not belong to a unique plane and consequently, several homographies $\left\{\;H_{k}^{lut}\right\}$ should map the object’s points. In order to estimate the error of the mapped points, white-red landmarks were attached to some objects. In this case, the sensory rig was not moved; the objects were positioned at different distances within the depth of field of the system, and a total of 37 image samples were acquired. Figure 13 and Figure 14 show the results of the registration process for two image samples.

## 4. Results and Discussion

**Figure 15:**
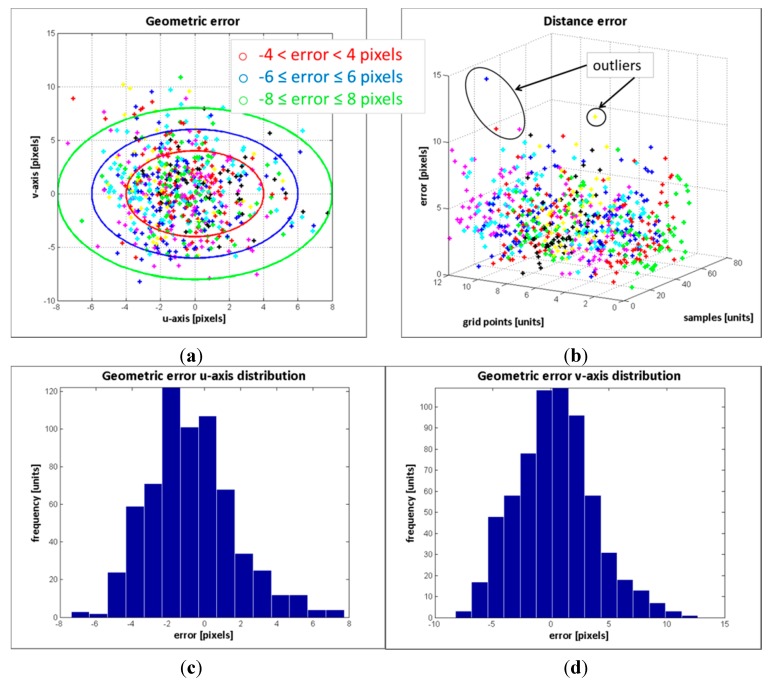
Error evaluation of the experimental tests corresponding to group #1. (**a**) geometric error; (**b**) distance error; (**c**) error distribution in *u*-axis; (**d**) error distribution in *v*-axis.

**Figure 16:**
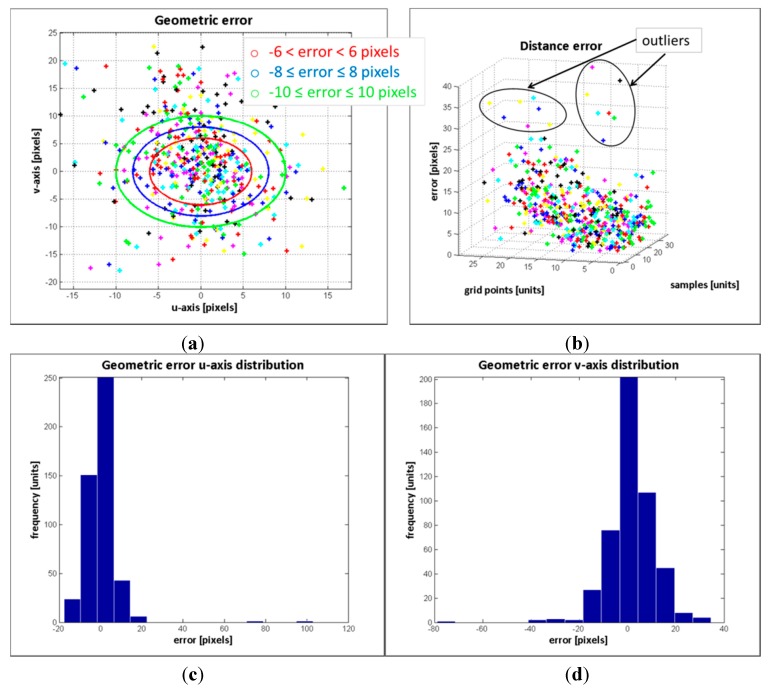
Error evaluation of the experimental tests corresponding to group #2. (**a**) geometric error; (**b**) distance error; (**c**) error distribution in *u*-axis; (**d**) error distribution in *v*-axis.

Two series of experimental tests were conducted to evaluate the image registration procedure proposed in this work. In order to carry out a quantitative assessment of the error, white-red landmarks were attached to the objects of interest. Then, ground control points were selected from the RGB and ToF image pairs, and the geometric and distance errors were computed. The results of the error distribution are detailed in Table 4, while in Figure 15 and Figure 16 the results of the geometric and distance error for each group of experiments are displayed. The errors in terms of RMSE are 0.2323 and 0.8353 for the first and second groups, respectively. 

**Table 4:** Results of the error distribution.

Error Distribution (pixels)	Group #1		Group #2	
	Error Percentage (%)			
	*u-Axis*	*v-Axis*	*u-Axis*	*v-Axis*
error<|4|	89.1	76.7	60.7	44.4
|4|≤error ≤ |6|	9.2	17.1	16.5	12.5
|6|<error ≤ |8|	2.1	3.8	6.9	11.3
|8|<error≤ |10|	0	2.4	6.1	6.7
error> |10|	0	0	9.8	25.1

The results from evaluating the estimated data on the RGB image show that the response of the registration method is quite promising, given that a large number of the mapped points have geometric errors less than 4 pixels on the RGB frame and the RMSE is quite low. Most of the erroneous data are due to the depth measurement variations and the flawed data selected as correspondence ground control points. These errors are more pronounced in the second group, mostly because of the object perspective which increases the flying pixels and the multi-path interference of the depth measurements. Despite the error, the reconstruction of the colour depth map shows the object’s edges are properly matched, as are as the object’s shapes.

**Figure 17:**
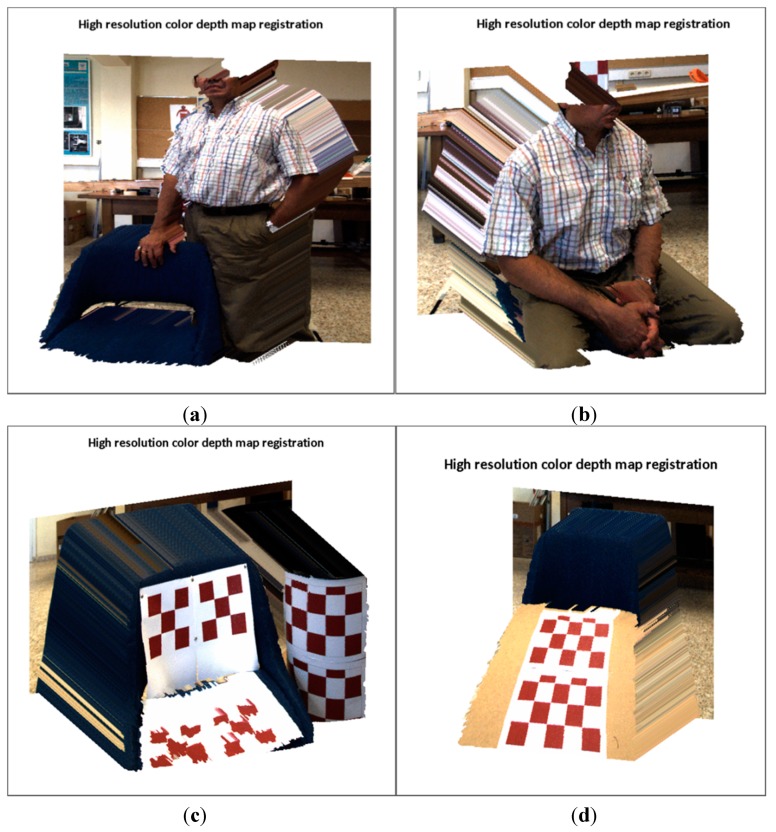
High resolution colour depth map reconstruction. (**a**) two volumetric objects placed at different distances from the sensory system; (**b**) an object with a large relief; (**c**) two curved objects; (**d**) a continuous surface which is slanted with respect to the cameras axis.

In conclusion, it is evident that the noise in the depth measurements is a drawback for computing the registration. Nonetheless, the proposed method has shown to be robust enough to deal with variations within ±12 mm. The object distance is also a relevant issue, since the sensing of edges, borders and dimensions of objects are altered because of the low ToF camera resolution. In addition due to the large difference in the camera resolution, when mapping points from the ToF to the RGB image, there are approximately 10 pixels of unmapped points between adjacent mapped points. Nevertheless, the proposed method provides a labelled RGB image (LRGB), which corresponds to the entries *k* of the homographies $\;H_{k}^{lut}$ used to transfer the data, meaning it is then possible to classify the unmapped points by assigning them a $\;H_{k}^{lut}$. Up to now, the proposed method was able to put colour on the depth map. Now, by using the full classified LRGB images, the method is also capable of assigning depth to the colour information. Thus a high-resolution colour-depth map is computed by transferring the RGB data to the ToF frame such that$\;xmap_{i}^{ToF}=H_{k}^{{lut}^{-1}}x_{i}^{RGB}$. In Figure 17, the results of the rendering of the high resolution colour depth map 5 Megapixels in size for four random scenes are depicted. The visual results show a satisfactory performance of the proposed method. In order to test the method, these scenes comprise non-planar objects which are clearly explained with several planes. For instance, the cylinder, the chair, or the angled board are continuous surface, and there are properly mapped, without any presence of discontinuity on its surfaces. The object shape and edges presented almost no difficulties or artifacts, and so far, no enhancement algorithm has been implemented.

## 5. Conclusions

In this research a framework for automatic image registration of ToF and RGB images is presented. The proposed framework is capable of registering images with non-common features and dealing with fluctuations in depth measurements. The method is based on a depth-dependent homography lookup table (*Hlut)*. By this means, the 3D world is parametrized in *n*-planes which correspond to the entries of the *Hlut*. Hence, point transformation between views is reliant on the distance from the objects to the sensory system, this being a non-feature based method. Since the method relies on planar projective transformation, the computation load is very low, making it suitable for near real-time applications.

The experimental results show that the proposed solution exhibits a satisfactory performance in terms of both visual quality and RMSE. The method normally maps points with an error of less than 4 pixels, measured on the RGB frame, which is a small error considering the RGB camera resolution. These errors represent slight distortions of the mapped points at working distances within 300–1300 mm. The procedure is capable of computing a low resolution colour depth map together with a labelled RGB image (LRGB). The values of the LRGB correspond to the homographies $\left\{\;H_{k}^{lut}\right\}$ used for transferring the data. Since there is a large difference in the camera resolutions, within adjacent mapped points on the RGB image, several coloured points remain unmapped. This labelled image is intended to be used for matching the unmapped points on the RGB image. In this work, a nearest neighbourhood algorithm was applied to create a mask of $\;H_{k}^{lut}$ on the RGB frame. Then, the high resolution colour depth map was straightforward computed by mapping points from the RGB to the ToF. Complex scenes with large texture variations, curved objects and large continuous surfaces were evaluated. The obtained visual results demonstrate that the depth-dependent *Hlut* method is capable of mapping these non-planar objects without any presence of discontinuity on their surfaces. Hence, the transition between homographies was successfully achieved. In conclusion, the computation of a high resolution colour dense map is possible and the loss of colour information is avoided. 

Regarding the response to noise in the depth estimations, the proposal of this work has shown better results than the response of the standard method for depth map registration. Since the *Hlut* method relies on discretized ranges of depth values, the fluctuations on the depth measurements are assumed by the proposed method. On contrary, the standard method directly depends on the depth value for transforming the data between two cameras.

For some robotic applications, the results presented in this work are accurate enough. Nevertheless, other applications might require high-quality and high-accuracy colour depth maps. In future research, more sophisticated algorithms for edge and depth measurement enhancement, as well as for the detection and removal of the outliers, will be investigated. Essentially, the labelled image was intended for further implementations of these algorithms. By means of the combination of the labelled information, the depth values and the texture of the RGB image, smart and guided algorithms can be adopted. 

The proposed framework is intended to be applied with other sensors such as thermal cameras, SWIR cameras, multispectral systems, and so on. For that purpose, an extensive and detailed description of the procedure for the depth-dependent *Hlut* method implementation is presented in this work.
